# A meta-analysis of genome-wide association studies of follicular lymphoma

**DOI:** 10.1186/1471-2164-13-516

**Published:** 2012-10-01

**Authors:** Christine F Skibola, Lucia Conde, Jia-Nee Foo, Jacques Riby, Keith Humphreys, Fenna CM Sillé, Hatef Darabi, Sylvia Sanchez, Henrik Hjalgrim, Jianjun Liu, Paige M Bracci, Karin E Smedby

**Affiliations:** 1 School of Public Health, Division of Environmental Health Sciences, University of California, Berkeley, 94720, CA, USA; 2 Human Genetics, Genome Institute of Singapore, A*STAR, 138673, Singapore; 3 Dept of Medical Epidemiology and Biostatistics, Karolinska Institutet, Stockholm, Sweden; 4 Department of Epidemiology Research, Statens Serum Institut, Copenhagen, Denmark; 5 Department of Epidemiology and Biostatistics, University of California, San Francisco, CA, 94143, USA; 6 Unit of Clinical Epidemiology, Dept of Medicine Solna, Karolinska Institutet, Stockholm, Sweden

**Keywords:** Follicular lymphoma (FL), Genome-wide association studies (GWAS), Human leukocyte antigen (HLA), Meta-analysis

## Abstract

**Background:**

B-cell non-Hodgkin lymphoma represents a diverse group of hematological malignancies, of which follicular lymphoma (FL) is one of the most common subtypes. Family and epidemiological studies suggest an important genetic role in the etiology of FL. In recent genome-wide association studies (GWAS) of FL, several genetic susceptibility loci have been identified on chromosome 6p21.33 (rs6457327) and 6p21.32 (rs10484561, rs2647012) in the human leukocyte antigen class I and class II regions. To identify new genetic variants and further elucidate the genetic basis of FL, a meta-analysis was performed of the top 1000 SNPs associated with FL risk from two GWAS in the US, Denmark and Sweden (592 cases, 1541 controls), with independent validation in 107 cases and 681 controls.

**Results:**

rs9275517 and rs3117222 in the HLA class II region were validated and inversely associated with FL risk (rs9275517: OR = 0.63, 95% CI = 0.55-0.73, p = 4.03 × 10^-11^; rs3117222: OR = 0.66, 95% CI = 0.57-0.77, p = 1.45 × 10^-7^). rs9275517, which is in high linkage disequilibrium with rs2647012 (r2 = 0.9), was no longer associated with FL after conditioning on rs2647012. The rs3117222 association was independent of established FL SNPs, but not of the HLA-DPB1*0301 allele. Using publicly available gene expression profiles with matching genotype information, we found that rs3117222 also was significantly correlated with increased *HLA-DPB1* expression.

**Conclusions:**

By performing a meta-analysis of two GWAS of FL, we further validated the relevance of HLA-DPB1*0301 as a protective allele in the pathogenesis of FL. Moreover, the protective rs3117222 A allele correlated with increased levels of *HLA-DPB1*, suggesting a possible disease mechanism involving *HLA-DPB1* expression regulation. Our results add further support to the major role of HLA genetic variation in the pathogenesis of FL.

## Background

Follicular lymphoma (FL) is a B-cell neoplasm that represents the second most common form of B-cell non-Hodgkin lymphoma (NHL). An important role for inherited genetic susceptibility for FL has been supported by recent genome-wide association studies (GWAS) where three independent susceptibility alleles have been identified in the HLA class I (rs6457327) [1] and class II (rs10484561, rs2647012) regions [2,3]. Follow-up HLA sequencing studies revealed that rs10484561 is in complete linkage disequilibrium (LD) with the DRB1*0101-DQA1*0101-DQB1*0501 extended haplotype, and rs2647012 is highly correlated with DRB1*15-DQA1*01-DQB1*06 [4]. An independent inverse association with FL risk also was found for DPB1*0301[4]*.* Because previous GWAS only attempted to validate the top 40 variants associated with FL [2,3], here we conducted a meta-analysis of the top 1000 SNPs from existing GWAS data in 592 FL cases and 1541 controls from Denmark/Sweden (SCALE) and the San Francisco Bay Area (SF-NHL2) to identify new genetic variants and further elucidate the genetic basis of FL. Validation genotyping of associated SNPs was conducted in 107 FL cases and 681 controls from an independent NHL case–control study population (SF-NHL1). The effect of validated SNP genotypes on gene expression levels also was investigated using publicly available microarray data.

## Results and discussion

After excluding the SNPs previously tested for validation in the two GWAS [2,3], 62 SNPs located in 20 independent loci were associated with FL at a *p*-value threshold of 1×10^−4^ in the random-effects meta-analysis (Additional file 1: Table S1). For each independent locus, we selected the SNP with the lowest p-value and removed markers in LD. Of the remaining SNPs, only those with evidence of association from at least a secondary marker in the GWAS were taken forward to validation. Among the 11 independent SNPs selected for validation in the SF NHL1 study, two SNPs, rs9275517 and rs3117222, located on 6p21.32 in the HLA class II region, were validated in this third independent population (rs9275517: OR = 0.58, 95% CI: 0.37-0.92, FDR-adjusted p-value = 2.29 × 10^-3^; rs3117222: OR = 0.46, 95% CI: 0.29-0.73, FDR-adjusted p-value = 2.83 × 10^-3^, Additional file 2: Table S2). In the combined analysis of all three studies, rs9275517 (random-effects p-value = 4.03 × 10^-11^; *P*_heterogeneity_ = 0.76, I^2^ = 0%) and rs3117222 (random-effects p-value = 1.45 × 10^-7^; *P*_heterogeneity_ = 0.77, I^2^ = 0%) were associated with FL at a genome-wide significant level (*i.e*., *P* < 5.0 × 10^−7^; Table 1, Additional file 3: Table S3).

**Table 1:** Summary results for the validated follicular lymphoma associated single-nucleotide polymorphisms

**SNP**	**Gene(s) within 5 kb (hg18)**	**LOC (hg18)**	**A1/A2**	**SF-NHL2 GWAS**			**SCALE GWAS**			**SF-NHL1 Validation**			**Combined meta-analysis**			
				**Logistic p-value**	**Logistic OR (95% CI)**	**MAF ca/co**	**Logistic p-value**	**Logistic OR (95% CI)**	**MAF ca/co**	**Logistic p-value**	**Logistic OR (95% CI)**	**MAF ca/co**	P-value*	OR* (95% CI)	**Q**	**I**^**2**^
rs9275517	-	chr6 32782627	A/G	7.56E-04	0.67 (0.53- 0.85)	0.29/0.38	6.73E-07	0.63 (0.53-0.76)	0.37/0.48	2.32E-03	0.57 (0.39-0.82)	0.29/0.41	**4.03E-11**	0.63 [0.55-0.73]	**0.7596**	0% (0%-62.2%)
rs3117222	HLA-DPB1, Q30181	chr6 33168927	A/G	4.17E-03	0.67 (0.51- 0.88)	0.18/0.25	3.06E-04	0.68 (0.55-0.84)	0.19/0.26	5.72E-03	0.58 (0.39-0.85)	0.16/0.25	**1.45E-07**	0.66 [0.57-0.77]	**0.7673**	0% [0%-60.7%]

*P-values and ORs estimated from the meta-analysis were identical for the random- and fixed-effects models.

A1/A2 = minor/major alleles; Q = Cochran’s Q statistic; I^2^ = heterogeneity index.

To determine whether rs9275517 and rs3117222 are independently associated with FL risk, we adjusted our models for the previously identified FL-risk loci, rs10484561, rs2647012, and rs6457327 [1-3]. The results showed that rs9275517 was no longer associated with FL after conditioning on rs2647012 in any of the studies (p-value_SF_ = 0.31, p-value_SCALE_ = 0.63, Additional file 4: Table S4). Further, rs9275517 and rs2647012 are in high LD (r^2^ = 0.9 in HapMap-CEU), also suggesting that the signal observed for rs9275517 is not independent of the previously validated protective rs2647012 allele [3]. In contrast, rs3117222 maintained independent statistical significance when each SNP was fitted in the logistic regression model (p < 0.02, Additional file 4: Table S4). Because rs3117222 maps 6 kb downstream of the *HLA-DPB1* gene, we also tested the independence of rs3117222 with *HLA-DPB1* alleles including DPB1*0301, an allele inversely associated with FL based on HLA sequencing studies of the SF-NHL2 study population [4]. Haplotype analysis in the SF-NHL2 data showed a significant difference in the frequencies of the HLA-DPB1*0301-rs3117222 haplotype in cases versus controls (p-value = 9.00 × 10^-4^, Additional file 5: Table S5) and the LD analysis suggested that rs3117222 was in LD with HLA-DPB1*0301 (*r*^2^ = 0.24, D' = 0.96). When DPB1*0301 was included in the model, rs3117222 was no longer associated with FL (p-value = 0.33), indicating that although rs3117222 is independent of previously reported FL-associated SNPs, its influence on FL risk cannot be delineated from the protective HLA-DPB1*0301 allelic association. Nonetheless, through rs3117222, this study provides the first validation of the *HLA-DPB1* locus as protective in the pathogenesis of FL in two additional independent studies (SCALE and SF-NHL1).

**Figure 1:**
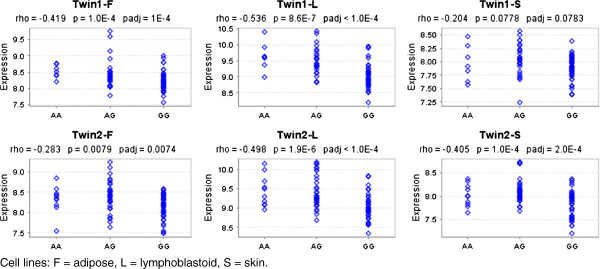
**Correlation of rs3117222 genotypes with *HLA-DPB1 *expression in the MuTHER dataset.** Cell lines: F = adipose, L = lymphoblastoid, S = skin.

**Figure 2:**
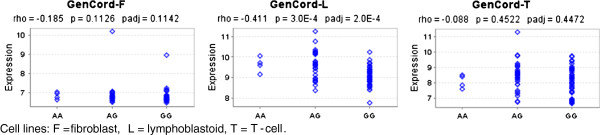
**Correlation of rs3117222 genotypes with *HLA-DPB1 *expression in the Gen Cord dataset.** Cell lines: F = fibroblast, L = lymphoblastoid, T = T-cell.

To explore whether rs3117222 affects *HLA-DPB1* gene expression, we used two publicly available mRNA expression datasets from the MuTHER [5] and Gen Cord [6] projects. We found a strong correlation between the protective rs3117222 A allele and increased *HLA-DPB1* expression in all lymphoblastoid cell lines in both datasets (Figures 1 and 2), indicating that enhanced *HLA-DPB1* expression may play a protective role in the etiology of FL. This hypothesis is in line with previous studies where reduced HLA class II expression on Hodgkin Reed–Sternberg cells and diffuse large B-cell lymphoma (DLBCL) tumor cells has been associated with poor survival for classical HL [7] and DLBCL [8], respectively. Based on a recent HLA sequencing study of FL [4], we showed that the protective versus deleterious *HLA-DPB1* alleles possess oppositely charged glutamic acid rather than lysine residues at position 69 in binding pocket 4, factors that may influence peptide affinities for *HLA-DPB1*[9]. The present study suggests that an additional mechanism involving effects on *HLA-DPB1* expression may also influence FL risk. Further studies will be needed to confirm and clarify the specific mechanisms through which reduced *HLA-DPB1* expression may contribute to deregulated cellular processes that drive FL and its progression.

## Conclusions

In the present study, we found that rs3117222 was inversely associated with FL risk independent of previously published FL SNPs, but not independent of the HLA-DPB1*0301 allele. Elevated levels of *HLA-DPB1* transcripts found in the presence of the protective rs3117222 A allele suggest that changes in *HLA-DPB1* expression may be involved in the etiology of FL. These findings emphasize the important role of *HLA* genetic variation in FL disease etiology and the need for further studies to clarify the mechanisms through which HLA class II expression contributes to FL pathogenesis.

## Methods

### Description of GWAS subjects, genotyping and statistical analyses

#### SF-NHL2 GWAS

Full details of the study design and methods, genotyping, quality control and statistical analyses have been described elsewhere [2]. A total of 312,768 markers genotyped in 213 FL cases and 750 controls passed our quality control criteria and were used for genome-wide association analysis. Population stratification and cryptic relatedness were tested and corrected for as described previously [2], resulting in a final inflation factor λ = 1.04. Associations with FL were tested using a Cochran-Armitage trend test in PLINK 1.07 [10]. Odd ratios (OR) and 95% confidence intervals (95% CI) were calculated using the mid-p method from the epitools package in R [11].

#### SCALE GWAS

Full details of the study design and methods, genotyping, quality control and statistical analyses have been published [3]. The final analysis included 298,680 genetic variants available for 379 cases and 791 controls. Study subjects with evidence of cryptic family relationships or outliers in terms of population stratification were removed as described previously [3], resulting in a final inflation factor λ = 1.028. Wald tests, treating minor allele counts as continuous covariates were used to test for association.

#### Validation study (SF-NHL1)

Full details of this population-based San Francisco Bay Area case–control study of NHL (1,591 cases, 2,515 controls) have been published [12,13]. Here, a subset of HIV-negative, non-Hispanic white individuals (107 FL cases, 681 controls) were used for validation.

The SF-NHL1 and SF-NHL2 study protocols were approved by the UCSF ethics review committee (Nos. 10–03698 and 10–00629, respectively), and the SCALE study was approved by the Ethical Review Board at Karolinska Institutet, Stockholm, Sweden (Dnr 99–154, 2007/624-32). All study participants provided informed consent.

### Statistical analysis

#### Meta-analysis of the SF-NHL2 and SCALE GWAS

We selected for meta-analysis the top 1000 SNPs most significantly associated with FL in the SF and SCALE GWAS. Among these SNPs, 13 (rs9275572, rs10484561, rs6457617, rs2858331, rs3763313, rs7755224, rs1493202, rs2857106, rs2157051, rs12529049, rs10511017, rs587791 and rs3130617) were present in the top 1000 of both GWAS. Selected SCALE GWAS SNPs that were not genotyped or did not pass direct genotyping quality control in the SF study were imputed using BEAGLE 3.0.3 [14] with haplotype data from HapMap phase II-CEU as a reference. Similarly, selected SF GWAS SNPs that were not genotyped in the SCALE study were imputed from the 1000 Genomes pilot1 CEU (August 2009 release) and the HapMap Phase II release 22 CEU datasets using IMPUTEv1 [15]. ORs for each individual study were estimated using unconditional logistic regression under an additive model in PLINK. Before meta-analysis, genomic control (GC) was applied to each study by multiplying the standard error of the effect estimates by the square root of the study-specific inflation factor (*λ* = 1.04 in SF, *λ* = 1.03 in SCALE). ORs and GC-corrected standard errors were then combined in a meta-analysis under fixed- and random-effects inverse variance models using the metagen function from the meta package in R [16]. Heterogeneity across studies was tested with the Cochran’s Q test and quantified with the I^2^ heterogeneity index.

#### Validation - SNP selection and analysis

We selected for validation those SNPs with a random-effects p-value threshold <10^-4^ in the meta-analysis. To focus on newly associated loci not previously reported, the top 40 SNPs associated with FL from each GWAS were excluded from further validation, as these had been tested previously [2,3]. For the remaining markers, we used PLINK to group SNPs in LD based on genotypes from HapMap-CEU r28, resulting in 20 independent loci (r^2^ < 0.05). Eleven of these SNPs were taken forward to the validation stage based on 1) lowest meta-analysis p-value and 2) evidence of a secondary signal at each locus in the original GWAS. Association analyses were conducted using trend and logistic regression tests in PLINK 1.07. P-values were adjusted for multiple comparisons using the Benjamini-Hochberg FDR correction from the p-adjust function in R [17] and considered significant at a FDR adjusted p-value level = 0.05. ORs and 95% CI were calculated for the variant allele carriers using the epitools package. To estimate overall association in the discovery and validation phases, logistic regression ORs and standard errors for the validated SNPs were combined with GC-corrected estimates from the original GWAS in a meta-analysis under fixed- and random-effects models.

#### Statistical analysis adjusting for previously associated variants

Logistic regression analysis was conducted adjusting for the additive effects of FL-associated SNPs that were entered separately into the model as covariates using a 0,1,2 allele dosage coding. The same approach was used in the SF-NHL2 dataset to test for independent effects between SNPs and HLA alleles that were previously typed in 205 FL cases and 82 controls as part of the SF GWAS [4]. HLA alleles were coded as binary alleles where A = present and B = absent.

#### Linkage disequilibrium (LD) and haplotype analysis

LD metrics between SNPs were based on European samples (CEU) from HapMap release 28 [18]. LD between rs3117222 and *HLA-DPB1* alleles were based on genotype and HLA typing data from the SF GWAS study. Haplotype analyses were carried out with Haploview [19].

#### Gene expression analysis

Associations with gene expression were investigated using two publicly available datasets, one with 166 adipose, 160 skin, and 156 lymphoblastoid cell lines derived from a subset of healthy female twins of the MuTHER study [5], and a second dataset with three cell types (fibroblast, lymphoblastoid and T-cell) derived from umbilical cords of 75 Geneva GenCord individuals [6]. Correlation between SNPs and gene expression levels was assessed by the Spearman rank correlation test with p-values adjusted by permutations using the Genevar application [20].

## Abbreviations

CI: Confidence interval; DLBCL: Diffuse large B-cell lymphoma; FL: Follicular lymphoma; GWAS: Genome-wide association studies; HL: Hodgkin lymphoma; HLA: Human leukocyte antigen; LD: Linkage disequilibrium; NHL: Non-Hodgkin lymphoma; OR: Odds ratio; SNP: Single nucleotide polymorphism.

## Competing interests

The authors declare that they have no competing interests.

## Authors’ contributions

CFS, LC and KES designed and interpreted the overall study. CFS, LC and KES drafted the manuscript. LC analyzed data. CFS, LC, JR and PMB participated in the study design, genotyping and data analysis in the SF-NHL2 GWAS. J-NF, KH, HD, HH, JL and KES participated in the study design, genotyping and data analysis in the SCALE GWAS. FCMS and SS performed genotyping in the SF-NHL1 validation study. All authors have read and approved the final manuscript.

## Supplementary Material

Additional file 1 **Table S1.** List of single-nucleotide polymorphisms (SNPs) that were significantly (random effects p-value < 10^-4^) associated with risk of follicular lymphoma (FL) in the meta-analysis of 592 FL cases and 1541 controls from Denmark/Sweden (SCALE) and the San Francisco Bay Area (SF-NHL2) studies. **Table S2.** Results for the 11 single-nucleotide polymorphisms (SNPs) selected for validation in an independent follicular lymphoma case–control study from the SF Bay Area (SF-NHL1). **Table S3.** Meta-analysis of the combined GWAS and validation datasets for the 11 SNPs selected for validation. **Table S4.** Logistic regression results for the validated SNPs in the SF-NHL2 and SCALE GWAS. P-values were computed in a logistic regression model with and without adjustment for established FL-associated SNPs. **Table S5.** Case–control frequencies and association p-values for the most frequent HLA-DPB1/rs3117222 haplotypes in the SF-NHL2 population. Click here for file
