# Supplementary material for: A meta-analysis of genome-wide association studies of follicular lymphoma
Source: BMC Genomics. 2012 Oct 1;13:516. doi: 10.1186/1471-2164-13-516 (PMC3534234; doi:10.1186/1471-2164-13-516)
Supplement: Additional file 1 — Table S1. List of single-nucleotide polymorphisms (SNPs) that were significantly (random effects p-value < 10-4) associated with risk of follicular lymphoma (FL) in the meta-analysis of 592 FL cases and 1541 controls from Denmark/Sweden (SCALE) and the San Francisco Bay Area (SF-NHL2) studies. Table S2. Results for the 11 single-nucleotide polymorphisms (SNPs) selected for validation in an independent follicular lymphoma case–control study from the SF Bay Area (SF-NHL1). Table S3. Meta-analysis of the combined GWAS and validation datasets for the 11 SNPs selected for validation. Table S4. Logistic regression results for the validated SNPs in the SF-NHL2 and SCALE GWAS. P-values were computed in a logistic regression model with and without adjustment for established FL-associated SNPs. Table S5. Case–control frequencies and association p-values for the most frequent HLA-DPB1/rs3117222 haplotypes in the SF-NHL2 population. [file 1471-2164-13-516-S1.doc]

**Supplementary Table 1**. List of single-nucleotide polymorphisms (SNPs) that were significantly (random effects p-value < 10^-4^) associated with risk of follicular lymphoma (FL) in the meta-analysis of 592 FL cases and 1541 controls from Denmark/Sweden (SCALE) and the San Francisco Bay Area (SF-NHL2) studies.

| **SNP** | **Gene(s) located <5kb away (Ensembl v48)** | **LOC (Ensembl v48)** | **A1/A2** | **Random-effects p-value** | **Random-effects  OR (95% CI)** | **Fixed-effects p-value** | **Fixed-effects  OR (95% CI)** | **I2** | **Q** |
| --- | --- | --- | --- | --- | --- | --- | --- | --- | --- |
| rs9275517 | - | 6-32782627 | A/G | 3.77E-09 | 0.65 (0.56-0.75) | 3.77E-09 | 3.77E-09 | 0% | 0.7209 |
| rs28605404 | - | 6-32677665 | G/A | 1.88E-08 | 1.76 (1.45-2.15) | 1.88E-08 | 1.88E-08 | 0% | 0.7406 |
| rs35465556 | - | 6-32556112 | C/T | 4.41E-08 | 1.75 (1.43-2.13) | 4.41E-08 | 4.41E-08 | 0% | 0.7403 |
| rs35998847 | - | 6-32774975 | A/G | 6.08E-08 | 1.68 (1.39-2.03) | 6.08E-08 | 6.08E-08 | 0% | 0.8279 |
| rs35571839 | - | 6-32620591 | T/C | 9.77E-08 | 1.67 (1.38-2.02) | 9.77E-08 | 9.77E-08 | 0% | 0.8302 |
| rs28895171 | HLA-DRB9 | 6-32530999 | A/G | 1.13E-07 | 1.62 (1.36-1.94) | 1.13E-07 | 1.13E-07 | 0% | 0.7931 |
| rs28895187 | HLA-DRB9 | 6-32532358 | A/G | 1.35E-07 | 1.62 (1.35-1.94) | 1.35E-07 | 1.35E-07 | 0% | 0.7721 |
| rs9274614 | ENSG00000214833, HB25_HUMAN | 6-32743824 | C/G | 1.37E-07 | 0.61 (0.51-0.73) | 1.37E-07 | 1.37E-07 | 0% | 0.8179 |
| rs13198610 | HLA-DRB9 | 6-32533650 | T/G | 1.41E-07 | 1.62 (1.35-1.93) | 1.41E-07 | 1.41E-07 | 0% | 0.7669 |
| rs34217071 | - | 6-32703020 | A/C | 2.92E-07 | 1.66 (1.37-2.02) | 2.92E-07 | 2.92E-07 | 0% | 0.8989 |
| rs28895078 | - | 6-32525869 | G/C | 3.43E-07 | 1.60 (1.33-1.91) | 3.43E-07 | 3.43E-07 | 0% | 0.9626 |
| rs9275245 | - | 6-32768921 | A/G | 3.62E-07 | 0.69 (0.60-0.80) | 3.62E-07 | 3.62E-07 | 0% | 0.9047 |
| rs28895103 | - | 6-32527442 | A/G | 3.70E-07 | 1.60 (1.33-1.91) | 3.70E-07 | 3.70E-07 | 0% | 0.5748 |
| rs2894253 | C6orf10 | 6-32453518 | G/T | 4.37E-07 | 1.68 (1.37-2.05) | 4.37E-07 | 4.37E-07 | 0% | 0.824 |
| rs3763313 | BTNL2 | 6-32484449 | C/A | 4.63E-07 | 1.52 (1.29-1.79) | 4.63E-07 | 4.63E-07 | 0% | 0.7365 |
| rs28530648 | - | 6-32635057 | C/A | 5.64E-07 | 1.63 (1.35-1.98) | 5.64E-07 | 5.64E-07 | 0% | 0.9227 |
| rs1493202 | - | 8-72068141 | A/C | 7.81E-07 | 1.41 (1.23-1.62) | 7.81E-07 | 7.81E-07 | 0% | 0.7087 |
| rs35954087 | - | 6-32548027 | T/C | 1.15E-06 | 1.64 (1.34-2.00) | 1.15E-06 | 1.15E-06 | 0% | 0.9574 |
| rs2857106 | TAP2, TA6P_HUMAN, HLA-DOB | 6-32895548 | G/A | 2.43E-06 | 1.51 (1.27-1.79) | 2.43E-06 | 2.43E-06 | 0% | 0.8528 |
| rs2157051 | - | 6-32766602 | C/T | 2.71E-06 | 0.64 (0.53-0.77) | 2.71E-06 | 2.71E-06 | 0% | 0.4075 |
| rs9273448 | ENSG00000198599, HB25_HUMAN | 6-32735725 | A/G | 3.15E-06 | 0.65 (0.54-0.78) | 3.15E-06 | 3.15E-06 | 0% | 0.6241 |
| rs9837401 | LPP | 3-189802758 | A/G | 3.90E-06 | 0.70 (0.60-0.81) | 3.90E-06 | 3.90E-06 | 0% | 0.8964 |
| rs28895095 | - | 6-32526826 | A/G | 4.18E-06 | 1.56 (1.29-1.89) | 4.18E-06 | 4.18E-06 | 0% | 0.5098 |
| rs3128917 | HLA-DPB1, Q30181_HUMAN | 6-33167974 | G/T | 5.83E-06 | 0.68 (0.57-0.80) | 5.83E-06 | 5.83E-06 | 0% | 0.9575 |
| rs3117222 | HLA-DPB1, Q30181_HUMAN | 6-33168927 | A/G | 5.83E-06 | 0.68 (0.57-0.80) | 5.83E-06 | 5.83E-06 | 0% | 0.9575 |
| rs12529049 | BTNL2 | 6-32465693 | T/C | 8.51E-06 | 1.51 (1.26-1.82) | 8.51E-06 | 8.51E-06 | 0% | 0.701 |
| rs587791 | - | 9-10308490 | T/G | 1.44E-05 | 1.47 (1.23-1.75) | 1.44E-05 | 1.44E-05 | 0% | 0.705 |
| rs3130617 | ENSG00000201207, APOM, BAT4, NP_067007.3 | 6-31735502 | C/T | 1.63E-05 | 0.69 (0.58-0.82) | 1.63E-05 | 1.63E-05 | 0% | 0.4957 |
| rs7756516 | A2ADX3_HUMAN | 6-32831895 | C/T | 1.72E-05 | 0.74 (0.64-0.85) | 1.72E-05 | 1.72E-05 | 0% | 0.4304 |
| rs16870123 | BTNL2 | 6-32467438 | A/G | 1.74E-05 | 1.49 (1.24-1.79) | 1.74E-05 | 1.74E-05 | 0% | 0.545 |
| rs9277565 | HLA-DPB1, Q30181_HUMAN | 6-33164875 | T/C | 1.76E-05 | 0.66 (0.55-0.80) | 1.76E-05 | 1.76E-05 | 0% | 0.9059 |
| rs4677602 | FOXP1 | 3-71462496 | T/C | 1.81E-05 | 1.37 (1.19-1.58) | 1.81E-05 | 1.81E-05 | 0% | 0.3926 |
| rs2691601 | SNX13 | 7-17841956 | C/T | 1.82E-05 | 0.74 (0.64-0.85) | 1.82E-05 | 1.82E-05 | 0% | 0.6592 |
| rs439205 | hsa-mir-219-1, SLC39A7, RING1, HSD17B8 | 6-33281820 | T/C | 1.88E-05 | 0.67 (0.56-0.81) | 1.88E-05 | 1.88E-05 | 0% | 0.9521 |
| rs10511017 | FOXP1 | 3-71492702 | C/T | 1.89E-05 | 1.42 (1.21-1.67) | 1.89E-05 | 1.89E-05 | 0% | 0.7218 |
| rs17423649 | C6orf10, BTNL2 | 6-32465111 | T/C | 2.14E-05 | 1.49 (1.24-1.80) | 2.14E-05 | 2.14E-05 | 0% | 0.6043 |
| rs35399661 | - | 6-32698968 | G/A | 2.35E-05 | 1.47 (1.23-1.75) | 2.35E-05 | 2.35E-05 | 0% | 0.8338 |
| rs12675271 | XKR9 | 8-71764563 | A/G | 2.60E-05 | 1.34 (1.17-1.53) | 2.60E-05 | 2.60E-05 | 0% | 0.535 |
| rs7192 | ENSG00000214861, HLA-DRA | 6-32519624 | T/G | 2.71E-05 | 0.73 (0.63-0.85) | 2.71E-05 | 2.71E-05 | 0% | 0.4218 |
| rs7013657 | XKR9 | 8-71800113 | G/A | 2.89E-05 | 1.34 (1.17-1.53) | 2.89E-05 | 2.89E-05 | 0% | 0.5488 |
| rs4669069 | - | 2-6488209 | T/C | 3.00E-05 | 0.74 (0.65-0.85) | 3.00E-05 | 3.00E-05 | 0% | 0.4644 |
| rs1524776 | SNX13 | 7-17838618 | C/A | 3.10E-05 | 0.74 (0.65-0.85) | 3.10E-05 | 3.10E-05 | 0% | 0.5211 |
| rs9859586 | FOXP1 | 3-71479168 | A/G | 3.77E-05 | 1.38 (1.18-1.60) | 3.77E-05 | 3.77E-05 | 0% | 0.3504 |
| rs2168802 | SLIT2 | 4-20194981 | T/C | 3.87E-05 | 0.70 (0.59-0.83) | 3.87E-05 | 3.87E-05 | 0% | 0.3228 |
| rs7638193 | FOXP1 | 3-71469750 | T/C | 3.92E-05 | 0.74 (0.65-0.86) | 3.68E-05 | 3.92E-05 | 0.60% | 0.3159 |
| rs2983478 | - | 14-94433843 | C/T | 4.01E-05 | 0.74 (0.65-0.86) | 4.01E-05 | 4.01E-05 | 0% | 0.5183 |
| rs9268832 | HLA-DRB9 | 6-32535767 | T/C | 4.32E-05 | 0.74 (0.64-0.85) | 4.32E-05 | 4.32E-05 | 0% | 0.377 |
| rs11994702 | - | 8-130913582 | A/C | 4.35E-05 | 0.65 (0.53-0.80) | 4.35E-05 | 4.35E-05 | 0% | 0.5403 |
| rs4937362 | - | 11-127997949 | T/C | 4.91E-05 | 1.33 (1.16-1.52) | 4.91E-05 | 4.91E-05 | 0% | 0.7399 |
| rs719654 | - | 6-32860117 | T/C | 5.04E-05 | 1.40 (1.19-1.65) | 5.04E-05 | 5.04E-05 | 0% | 0.7254 |
| rs1350400 | SLIT2 | 4-20222728 | G/A | 5.81E-05 | 1.55 (1.25-1.92) | 5.81E-05 | 5.81E-05 | 0% | 0.8109 |
| rs423285 | AJAP1 | 1-4730936 | G/A | 5.86E-05 | 0.70 (0.59-0.84) | 5.86E-05 | 5.86E-05 | 0% | 0.9625 |
| rs4677035 | FOXP1 | 3-71454424 | A/C | 5.96E-05 | 1.36 (1.17-1.58) | 5.96E-05 | 5.96E-05 | 0% | 0.36 |
| rs1497482 | - | 4-55491888 | G/A | 6.09E-05 | 1.33 (1.16-1.52) | 6.09E-05 | 6.09E-05 | 0% | 0.7128 |
| rs7203231 | AP1G1 | 16-70394978 | T/G | 6.30E-05 | 1.34 (1.16-1.54) | 6.30E-05 | 6.30E-05 | 0% | 0.5244 |
| rs2253812 | - | 10-119669249 | T/C | 6.40E-05 | 1.49 (1.23-1.82) | 6.40E-05 | 6.40E-05 | 0% | 0.3887 |
| rs2857597 | BAT2, AIF1 | 6-31692979 | A/T | 6.59E-05 | 0.71 (0.60-0.84) | 6.59E-05 | 6.59E-05 | 0% | 0.534 |
| rs2438709 | ARID5A | 2-96563421 | T/C | 7.41E-05 | 0.69 (0.58-0.83) | 7.41E-05 | 7.41E-05 | 0% | 0.5718 |
| rs3098859 | - | 8-72027586 | A/G | 8.21E-05 | 0.76 (0.66-0.87) | 8.21E-05 | 8.21E-05 | 0% | 0.3403 |
| rs6903608 | HLA-DRB9 | 6-32536263 | C/T | 9.08E-05 | 0.73 (0.62-0.85) | 9.08E-05 | 9.08E-05 | 0% | 0.6035 |
| rs1833619 | - | 19-13508830 | A/G | 9.74E-05 | 1.35 (1.16-1.57) | 9.74E-05 | 9.74E-05 | 0% | 0.4917 |
| rs17061325 | - | 5-162580043 | G/A | 9.88E-05 | 1.80 (1.34-2.42) | 5.00E-05 | 9.88E-05 | 7.80% | 0.2978 |

A1/A2 = minor/major alleles

**Supplementary Table 2**. Results for the 11 single-nucleotide polymorphisms (SNPs) selected for validation in an independent follicular lymphoma case-control study from the SF Bay Area (SF-NHL1).

| **SNP** | **Gene(s) located <5kb away (Ensembl48)** | **LOC (Ensembl v48)** | **A1/A2** | **MAF**  **ca/co** | **HWE** | **OR (95% CI)** | **Trend p-value** | **FDR-adjusted p-value** |
| --- | --- | --- | --- | --- | --- | --- | --- | --- |
| rs9275517 | - | 6:32782627 | A/G | 0.29/0.41 | 0.1482 | 0.58 (0.37-0.92) | 2.08E-03 | 2.29E-02 |
| rs3117222 | HLA-DPB1, Q30181_HUMAN | 6:33168927 | T/C | 0.16/0.25 | 0.4097 | 0.46 (0.29-0.73) | 5.14E-03 | 2.83E-02 |
| rs11994702 | - | 8:130913582 | A/C | 0.12/0.17 | 0.1173 | 0.62 (0.37-1.00) | 8.47E-02 | 3.11E-01 |
| rs2691601 | SNX13 | 7:17841956 | C/T | 0.51/0.47 | 0.7564 | 1.22 (0.77-1.99) | 1.87E-01 | 4.18E-01 |
| rs1493202 | - | 8:72068141 | T/G | 0.46/0.51 | 0.09788 | 0.81 (0.51-1.32) | 1.90E-01 | 4.18E-01 |
| rs12529049 | BTNL2 | 6:32465693 | T/C | 0.17/0.14 | 1 | 1.25 (0.79-1.94) | 3.05E-01 | 5.58E-01 |
| rs2168802 | SLIT2 | 4:20194981 | T/C | 0.19/0.21 | 0.7249 | 0.85 (0.54-1.32) | 4.68E-01 | 7.35E-01 |
| rs9837401 | LPP | 3:189802758 | A/G | 0.35/0.33 | 0.7868 | 0.97 (0.63-1.50) | 5.92E-01 | 7.45E-01 |
| rs4677602 | FOXP1 | 3:71462496 | T/C | 0.36/0.34 | 0.6581 | 1.14 (0.74-1.77) | 6.49E-01 | 7.45E-01 |
| rs1350400 | SLIT2 | 4:20222728 | C/T | 0.10/0.11 | 0.6841 | 0.89 (0.51-1.49) | 6.78E-01 | 7.45E-01 |
| rs587791 | - | 9:10308490 | T/G | 0.19/0.19 | 1 | 1.14 (0.73-1.75) | 8.19E-01 | 8.19E-01 |

A1/A2 = minor/major alleles. Odd ratios (OR) and 95% confidence intervals (95% CI) were calculated for the variant allele carriers (homozygous or heterozygous versus homozygous common allele carriers). FDR-adjusted=adjusted for multiple comparisons using the Benjamini-Hochberg False Discovery Rate (FDR) correction

**Supplementary Table 3**. Meta-analysis of the combined GWAS and validation datasets for the 11 SNPs selected for validation.

| **SNP** | **LOC (Ensembl v48)** | **A1/A2** | **Random-effects p-value** | **Random-effects  OR (95% CI)** | **Fixed-effects p-value** | **Fixed-effects  OR (95% CI)** | **Q** | **I2** |
| --- | --- | --- | --- | --- | --- | --- | --- | --- |
| rs9275517 | 6:32782627 | A/G | 4.03E-11 | 0.63 (0.55-0.73) | 4.03E-11 | 0.63 (0.55-0.73) | 0.7596 | 0% [0%; 62.2% |
| rs3117222 | 6:33168927 | A/G | 1.45E-07 | 0.66 (0.57-0.77) | 1.45E-07 | 0.66 (0.57-0.77) | 0.7673 | 0% [0%; 60.7% |
| rs11994702 | 8:130913582 | A/C | 9.33E-06 | 0.66 (0.54-0.79) | 9.33E-06 | 0.66 (0.54-0.79) | 0.8257 | 0% [0%; 45.7% |
| rs2691601 | 7:17841956 | C/T | 2.94E-01 | 0.86 (0.64-1.14) | 1.01E-03 | 0.81 (0.71-0.92) | 0.0087 | 78.9% [32.6%; 93.4% |
| rs1493202 | 8:72068141 | A/C | 2.26E-01 | 1.21 (0.89-1.64) | 8.99E-05 | 1.28 (1.13-1.45) | 0.0043 | 81.7% [43.2%; 94.1% |
| rs12529049 | 6:32465693 | T/C | 7.92E-06 | 1.46 (1.24-1.72) | 7.92E-06 | 1.46 (1.24-1.72) | 0.5891 | 0% [0%; 80.3% |
| rs2168802 | 4:20194981 | T/C | 7.38E-05 | 0.73 (0.62-0.85) | 5.17E-05 | 0.73 (0.62-0.85) | 0.3582 | 2.6% [0%; 89.9% |
| rs9837401 | 3:189802758 | A/G | 6.81E-02 | 0.79 (0.62-1.02) | 8.44E-05 | 0.76 (0.66-0.87) | 0.0459 | 67.5% [0%; 90.6% |
| rs4677602 | 3:71462496 | T/C | 7.35E-04 | 1.30 (1.12-1.51) | 4.29E-05 | 1.31 (1.15-1.50) | 0.2759 | 22.3% [0%; 91.9% |
| rs1350400 | 4:20222728 | G/A | 3.11E-02 | 1.37 (1.03-1.83) | 4.09E-04 | 1.42 (1.17-1.73) | 0.1419 | 48.8% [0%; 85.1% |
| rs587791 | 9:10308490 | T/G | 1.13E-03 | 1.37 (1.13-1.66) | 5.48E-05 | 1.38 (1.18-1.62) | 0.254 | 27% [0%; 92.4% |

A1/A2 = minor/major alleles

**Supplementary Table 4.** Logistic regression results for the validated SNPs in the SF-NHL2 and SCALE GWAS. P-values were computed in a logistic regression model with and without adjustment for established FL-associated SNPs.

| **SNP** | **GWAS study** | ***P*-value** | ***P*-value adjusted for rs10484561** | ***P*-value adjusted for rs6457327** | ***P*-value adjusted for rs2647012** |
| --- | --- | --- | --- | --- | --- |
| rs9275517 | SF-NHL2 | 7.56E-04 | 3.11E-02 | 1.09E-03 | 3.15E-01 |
|  | SCALE* | 6.73E-07 | 6.30E-05 | 7.55E-07 | 6.29E-01 |
| rs3117222 | SF-NHL2 | 4.17E-03 | 5.98E-03 | 2.72E-03 | 1.62E-02 |
|  | SCALE | 3.06E-04 | 8.02E-04 | 2.40E-04 | 3.61E-03 |

*rs9275517 was imputed in the SCALE study

**Supplementary Table 5.** Case-control frequencies and association p-values for the most frequent HLA-DPB1/rs3117222 haplotypes in the SF-NHL2 population.

| Haplotype | Freq. | Case, Control Ratio Counts | Case,Control Frequencies | Chi Square | P Value |
| --- | --- | --- | --- | --- | --- |
| DPB1*0401:rs3117222_G | 0.427 | 178.0 : 232.0, 67.0 : 97.0 | 0.434, 0.409 | 0.314 | 0.5752 |
| DPB1*0201:rs3117222_G | 0.169 | 77.0 : 333.0, 20.0 : 144.0 | 0.188, 0.122 | 3.618 | 0.0572 |
| DPB1*0402:rs3117222_G | 0.114 | 45.7 : 364.3, 19.8 : 144.2 | 0.111, 0.121 | 0.098 | 0.7542 |
| DPB1*0301:rs3117222_A | 0.062 | 16.9 : 393.1, 18.9 : 145.1 | 0.041, 0.115 | 10.978 | 9.00E-04 |
| DPB1*0101:rs3117222_A | 0.051 | 19.0 : 391.0, 10.0 : 154.0 | 0.046, 0.061 | 0.523 | 0.4696 |
| DPB1*1301:rs3117222_G | 0.031 | 17.0 : 393.0, 1.0 : 163.0 | 0.041, 0.006 | 4.823 | 0.0281 |
| DPB1*1101:rs3117222_G | 0.024 | 10.0 : 400.0, 4.0 : 160.0 | 0.024, 0.024 | 0 | 1 |
| DPB1*1401:rs3117222_A | 0.021 | 6.0 : 404.0, 6.0 : 158.0 | 0.015, 0.037 | 2.758 | 0.0968 |
| DPB1*0601:rs3117222_A | 0.019 | 10.0 : 400.0, 1.0 : 163.0 | 0.024, 0.006 | 2.085 | 0.1487 |
| DPB1*1001:rs3117222_A | 0.016 | 8.0 : 402.0, 1.0 : 163.0 | 0.020, 0.006 | 1.366 | 0.2425 |
| DPB1*0501:rs3117222_A | 0.014 | 7.0 : 403.0, 1.0 : 163.0 | 0.017, 0.006 | 1.027 | 0.3109 |
| DPB1*1501:rs3117222_A | 0.01 | 3.0 : 407.0, 3.0 : 161.0 | 0.007, 0.018 | 1.364 | 0.2428 |
